# Supplementary material for: MircoRNA in Extracellular Vesicles from Patients with Pulmonary Arterial Hypertension Alters Endothelial Angiogenic Response
Source: Int J Mol Sci. 2022 Oct 8;23(19):11964. doi: 10.3390/ijms231911964 (PMC9570422; doi:10.3390/ijms231911964)
Supplement: Supplementary file 1 [file ijms-23-11964-s001.zip › Supplementary Figures.pdf]

Supplementary Figure S1

A

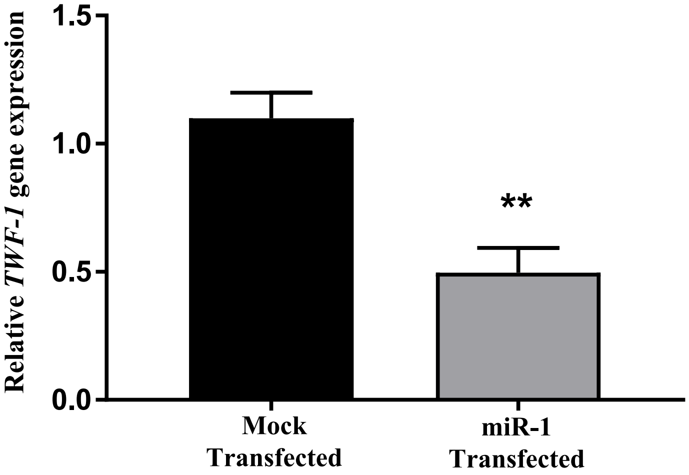

Figure S1: Relative TWF-1 gene expression to housekeeping GAPDH in hPAECs quantified by qRT-PCR. Data is given as mean with SD (n=3 per group). P-value by Student's t-test. ** P<0.01.

Supplementary Figure S2

A

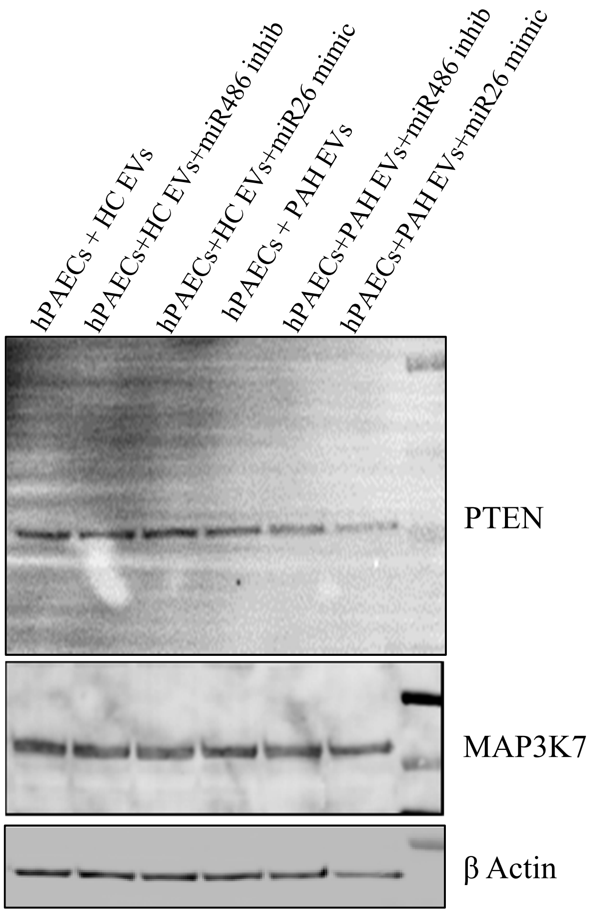

Figure S2: Representative immunoblots of PTEN and MAP3K7 along with housekeeping b-actin loading control in hPAECs preincubated with either HC or PAH EVs followed by transfection of miR inhibitor or mimic. (n=3).
